# Mitochondrial dynamics and respiration within cells with increased open pore cytoskeletal meshes

**DOI:** 10.1242/bio.029009

**Published:** 2017-11-14

**Authors:** David H. Jang, Sarah C. Seeger, Martha E. Grady, Frances S. Shofer, David M. Eckmann

**Affiliations:** 1 Department of Emergency Medicine, Division of Medical Toxicology and Critical Care Medicine, Perelman School of Medicine, University of Pennsylvania, 3620 Hamilton Walk, John Morgan Building Room 12, Philadelphia, PA 19104, USA; 2 Department of Materials Science and Engineering, University of Pennsylvania, Philadelphia, PA 19104, USA; 3 Department of Mechanical Engineering, University of Kentucky, 151 RGAN Building, Lexington, KY 40506, USA; 4 Department of Anesthesiology and Critical Care, Perelman School of Medicine, University of Pennsylvania, 3620 Hamilton Walk, John Morgan Building Room 27B, Philadelphia, PA 19104, USA; 5 Department of Bioengineering, University of Pennsylvania, Philadelphia, PA 19104, USA

**Keywords:** Cytoskeletal, Dynamics, Mitochondria, Respiration

## Abstract

The cytoskeletal architecture directly affects the morphology, motility, and tensional homeostasis of the cell. In addition, the cytoskeleton is important for mitosis, intracellular traffic, organelle motility, and even cellular respiration. The organelle responsible for a majority of the energy conversion for the cell, the mitochondrion, has a dependence on the cytoskeleton for mobility and function. In previous studies, we established that cytoskeletal inhibitors altered the movement of the mitochondria, their morphology, and their respiration in human dermal fibroblasts. Here, we use this protocol to investigate applicability of power law diffusion to describe mitochondrial locomotion, assessment of rates of fission and fusion in healthy and diseased cells, and differences in mitochondria locomotion in more open networks either in response to cytoskeletal destabilizers or by cell line. We found that mitochondria within fibrosarcoma cells and within fibroblast cells treated with an actin-destabilizing toxin resulted in increased net travel, increased average velocity, and increased diffusion of mitochondria when compared to control fibroblasts. Although the mitochondria within the fibrosarcoma travel further than mitochondria within their healthy counterparts, fibroblasts, the dependence on mitochondria for respiration is much lower with higher rates ofhydrogen peroxide production and was confirmed using the OROBOROS O2K. We also found that rates of fission and fusion of the mitochondria equilibrate despite significant alteration of the cytoskeleton. Rates ranged from 15% to 25%, where the highest rates were observed within the fibrosarcoma cell line. This result is interesting because the fibrosarcoma cell line does not have increased respiration metrics including when compared to fibroblast. Mitochondria travel further, faster, and have an increase in percent mitochondria splitting or joining while not dependent on the mitochondria for a majority of its energy production. This study illustrates the complex interaction between mitochondrial movement and respiration through the disruption of the cytoskeleton.

## Abstract (precis)

**Summary:** We assessed the effect of cytoskeletal inhibitors and the use of a calcium ionophore as an additional stressor on mitochondrial motility and bioenergetic function in fibroblasts and fibrosarcoma cells.

## INTRODUCTION

Mitochondrial organization and networking play an important role in the energetic function of the cell. The mitochondria often localize to areas of high energy demand such as the growth cones in neurons (Morris and Hollenbeck, 1993). The mitochondria in healthy cells often exhibit characteristic motility, which can be impaired in diseased cells. For example, in Parkinson's disease, mitochondria exhibit abnormal distribution along the axons as well as increased mitochondrial fusion with reduced motility (Perier and Vila, 2012; Yang and Lu, 2009). Similarly, in cancer, mitochondria exhibit abnormal motility, which may be, in part, due to their glycolytic dependency (Desai et al., 2013).

The mitochondria play a central role in cellular metabolism, where oxygen consumption through the electron transport system (ETS) is tightly coupled to ATP production and regulated by metabolic demands (Wallace et al., 2010; Chance and Williams, 1955; Brown, 1992, 1995). Mitochondrial respiration influences key processes such as oxidative phosphorylation, ion gradients, membrane potential, reactive oxygen species (ROS) generation such as hydrogen peroxide (H_2_O_2_), and heat dissipation. The measurement of mitochondrial respirometry reflects a dynamic state that contrasts with static measures of mitochondrial function such as enzyme levels and metabolites. Failure of the mitochondria in normal cells to use oxygen to sustain ATP production results in an energy deficit, which can impair cell function best measured with high-resolution respirometry (HRR) (Pesta and Gnaiger, 2012).

The mitochondria are dynamic organelles that adapt in response to environmental stresses or mutations (Giedt et al., 2012a,b; Benard et al., 2007). Dynamic processes include mitochondrial locomotion within the cell and changes in states of fusion and fission. The mobile mitochondria move independently of each other and can move in different directions. Directed long-range locomotion occurs both away from the nucleus (anterograde) and towards the center of the cell and nucleus (retrograde) (Frederick and Shaw, 2007). In addition to movement, the mitochondria also undergo fission and fusion. Fission (in which mitochondria divide) is essential for growing and dividing cells, as it populates them with adequate numbers of mitochondria. Fission can also occur when there is significant mitochondrial damage that will allow the cell to segregate the damaged portion. Fusion (in which mitochondria merge) is also critical as it can eliminate defects in mitochondria by allowing them to cross-complete one another. Fusion can therefore maximize oxidative capacity in response to toxic stress (Giedt et al., 2012a,b; Song et al., 2008).

The cytoskeletal architecture is responsible for a variety of critical cellular functions, including support for cell stiffness and transportation of organelles (Kandel et al., 2016, 2017). The primary structures of the cytoskeletal architecture include intermediate filaments, actin filaments and microtubules, of which the last two play an important role in mitochondrial dynamics and respiration. Our recent study demonstrated that alteration of the cytoskeletal structure directly impacted both mitochondrial morphology and respiration in fibroblasts (Kandel et al., 2015). Microtubule destabilization with nocodazole (noco) led to a decrease in routine respiration that intensified when the mitochondria were additionally challenged with elevated calcium levels through the use of calcium ionophore A23187 (A23), which has been shown to cause disruption of microtubules by itself (Belmadani et al., 2002). Actin depolymerization through cytochalasin D (CytD), however, only affected mitochondrial respiration under conditions of disturbed calcium homeostasis. A23 alone, despite having multiple effects on mitochondrial morphology as a byproduct of increasing levels of intercellular calcium, including causing an increase in the amount of cytoskeletal-associated actin (Sha'afi et al., 1986) and further causing actin filaments to become fragmented (Kuhne et al., 1993), did not affect mitochondrial respiration. Thus, our study demonstrates the complex interaction between cytoskeletal architecture and mitochondrial function. In this study, we explored the effects of cytoskeletal inhibitors on the interaction between mitochondrial bioenergetics and dynamics in both adherent human dermal fibroblasts and fibrosarcoma cells as a contrasting cell line. More specifically, we assessed the effect of cytoskeletal inhibitors and the use of A23 as an additional stressor on mitochondrial respiration and reactive oxygen species (ROS, indicated by hydrogen peroxide production) in both cell lines. We further evaluated the effect of these same conditions on mitochondrial dynamics by assessing mitochondrial net movement along with rates of fusion and fission events. Overall, this will help to increase our understanding of the complex interactions of the alterations of the cellular cytoarchitecture with mitochondrial bioenergetics and dynamics.

## RESULTS

### Mitochondrial locomotion and power law diffusion

**Fig. 1.**
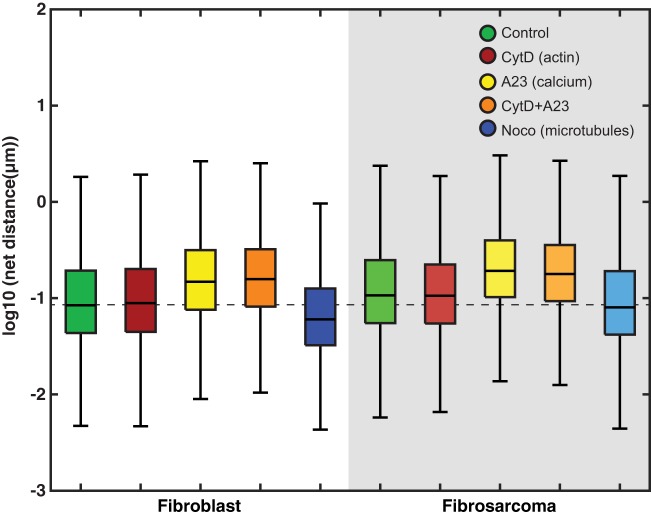
**Box plots showing net movement data from all groups.** Whiskers extend to 10th and 90th percentiles of data. The median of the control group is extended throughout the plot as a dashed line. Within the fibroblast group, cells exposed to both A23 and A23+CytD exhibited greater net movement when compared to both control and CytD-treated cells. In the fibroblasts exposed to noco, the mitochondria exhibited overall decreased net movement compared to all other conditions. Within the fibrosarcoma group, mitochondria exposed to A23 and A23+CytD exhibited greater net movement when compared to both fibroblast and fibrosarcoma controls.

Mitochondrial locomotion data were analyzed in three ways: net movement, total movement and power law diffusion. Net movement distributions do not follow a standard normal curve and thus an arithmetic average would not provide an appropriate summary statistic. Instead, we have chosen the geometric mean to represent the central tendency, as we have previously shown that the net distances follow a log-normal distribution (Kandel et al., 2015). The geometric mean of the mitochondrial net distances (nm) traveled by each cell condition is shown in Fig. 1 and *P*-value comparisons are in Table 1. Fig. S1 illustrates the lognormal distribution of net movement of select groups. Table S1 shows the *P*-values for total mitochondrial distances traveled along with average velocities.

**Table 1.**
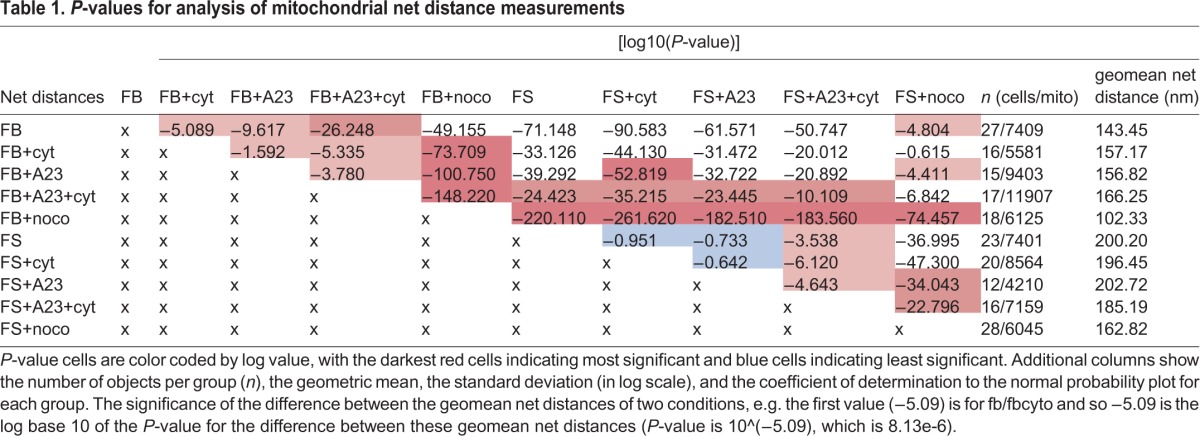
***P*-values for analysis of mitochondrial net distance measurements**

Notably, treating the fibroblast group with the calcium ionophore A23 or CytD caused an increase in mitochondrial net movement, which was exacerbated when treated with both. Fibroblast cells treated with noco, however, exhibited decreased mitochondrial net movement. Compared to the fibroblast group, mitochondria in the fibrosarcoma group displayed significantly higher net motion, which was unaffected by A23 and CytD treatments alone*.* Noco treatment decreased mitochondrial net motion in the fibrosarcoma group, following the same pattern seen in the fibroblast group. A23+CytD treatment caused a decrease in mitochondrial net motion in the fibrosarcoma group, contrary to what was found in fibroblasts. Total movement analysis mirrors the relationships found in net movement. Corresponding box plot and *P*-values are included in Fig. 1 and Table 1. An average velocity is calculated by using the average total distance of mitochondrial locomotion and dividing it by the total observation time. Our values ranged from 14.0 nm/s to 19.1 nm/s, and are within the range found by reports that mitochondria travel between 7 nm/s and 1000 nm/s (Li et al., 2004; Morris and Hollenbeck, 1995; Miller and Sheetz, 2004).

**Fig. 2.**
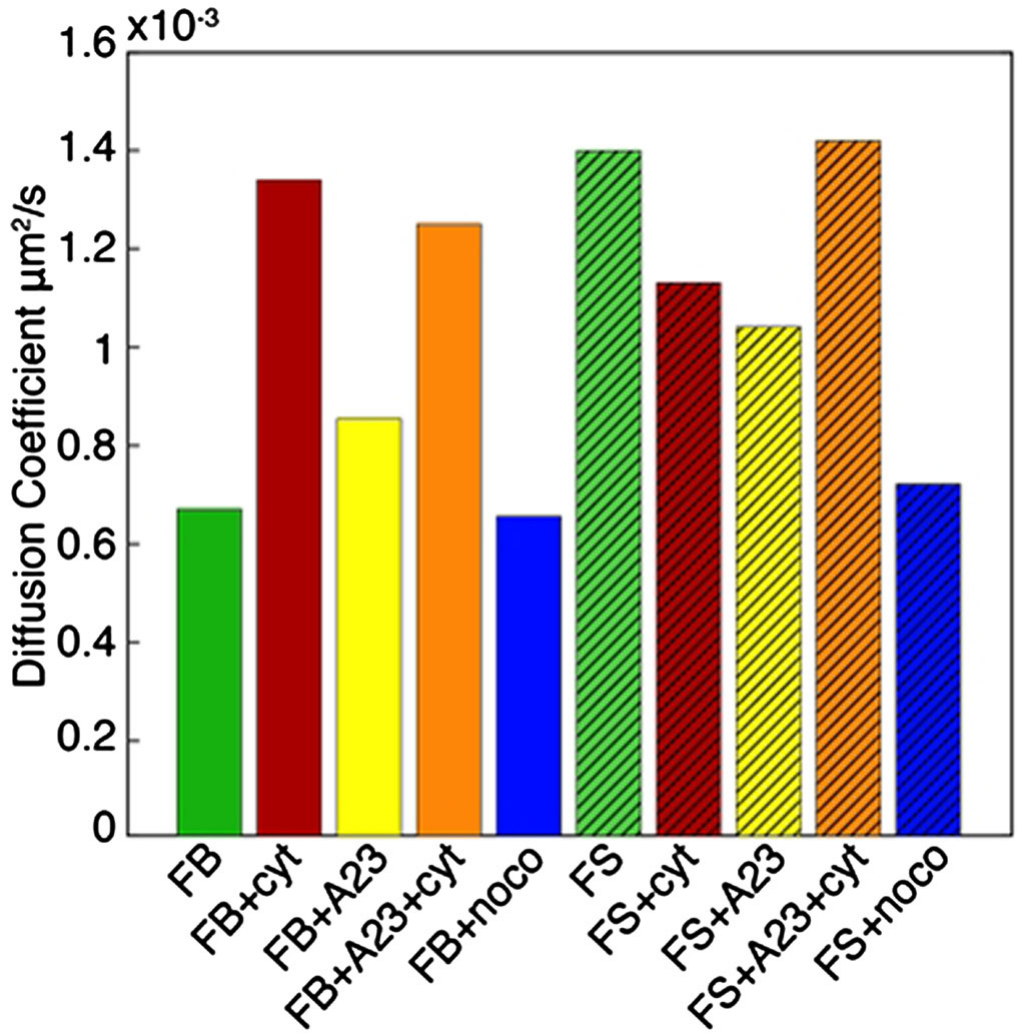
**Diffusion coefficients extracted from power law fit of mitochondria tracks for each cell type and drug condition.** Number of tracks varied for each condition but were always >700 tracks.

Prior work has been done in the area of applying a power law diffusive model to describe the locomotion of mitochondria in living cells as MSD=Dt^α^, where MSD is mean-squared displacement, D is dimensionality, t is time, and α is the power law exponent. We summarize this prior work in Table 2. For some, the type of locomotion was Brownian, having a linear relationship with MSD. For others, locomotion was categorized as subdiffusive (α<1) or directed (α>1). The diffusion coefficients reported are across different cell types and span several decades of magnitude: 1E-7 μm^2^/s to 6.5E-3 μm^2^/s. In our study, the diffusion coefficients of mitochondria within untreated or control fibroblasts were in the middle of this range, at 6.7E-4 μm^2^/s, and are shown in Fig. 2. Fig. S2 illustrates the distribution of power law diffusion exponents of mitochondria within fibroblast and fibrosarcoma controls. Mitochondria within untreated fibrosarcoma cells displayed a diffusion coefficient of 1.4E-3 μm^2^/s; double that of mitochondria within fibroblasts. Altering the actin network in the fibroblasts cell line with CytD treatment increased the diffusion coefficient to 1.3E-3 μm^2^/s, a value similar to that obtained with the fibrosarcoma cells. Based on our work with nanoparticle diffusion we believe the apparent differences in the diffusion of mitochondria within fibrosarcoma cells is due to the more open cytoskeletal network (Grady et al., 2017). This result is further emphasized by the increased mitochondria diffusion in fibroblast cells where the application of CytD has opened the cytoskeletal network.

**Table 2.**
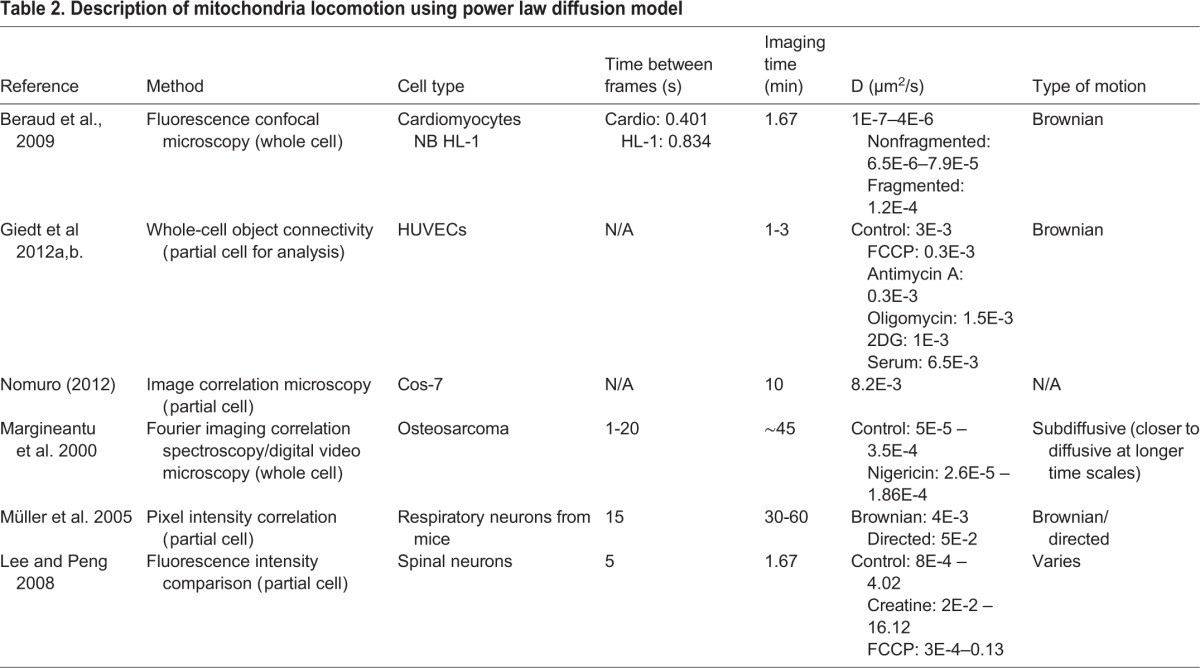
**Description of mitochondria locomotion using power law diffusion model**

Overall, comparison values of α derived from a power law fit indicated that mitochondria locomotion within fibroblasts and fibrosarcoma cells is subdiffusive, i.e., centered around α<1. Table S2 shows diffusion coefficients for each cell line and drug condition, with α ranging from 0.73 to 0.87. Mitochondria within the fibrosarcoma line, while still subdiffusive, exhibited a value of α closer to 1 than did mitochondria within the fibroblast line.

### Mitochondrial rates of fission and fusion equilibrate despite cytoarchitectural challenges

**Fig. 3.**
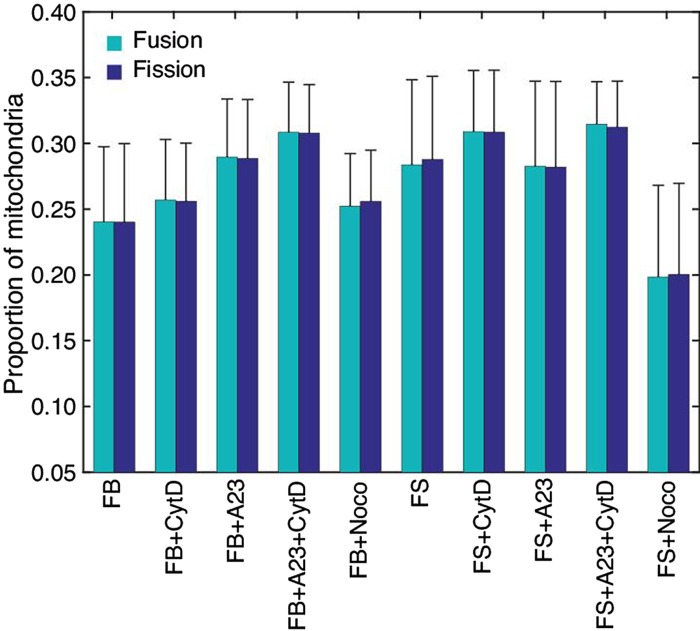
**Fraction of mitochondria undergoing fusion and fission events.** The number of fusion and fission events equilibrated within each exposure for both fibroblasts and fibrosarcoma cell types.

The percentages of mitochondria to undergo fission and fusion for each cell condition is displayed in Fig. 3. Fission and fusion rates calculated for cells in the same drug condition remarkably equilibrated to within 0.4% of each other. Overall, fission and fusion rates varied from a low value of 15%, which occurred within the fibrosarcoma line under noco treatment conditions, to a maximum value of 26%, which occurred within the fibrosarcoma line under A23+CytD treatment. The percentage of mitochondria that undergo fission is significantly higher for the fibrosarcoma group than the fibroblast control group, at values of 23.4% compared to 19.0%, respectively. Giedt et al. determined fission and fusion rates using an event/object*min calculation and found that this value also equilibrated after 30 min drug treatment exposure in vascular endothelial cells (Giedt et al., 2012a,b).

### Increase in mitochondrial locomotion in fibrosarcoma despite lower respiration metrics

**Fig. 4.**
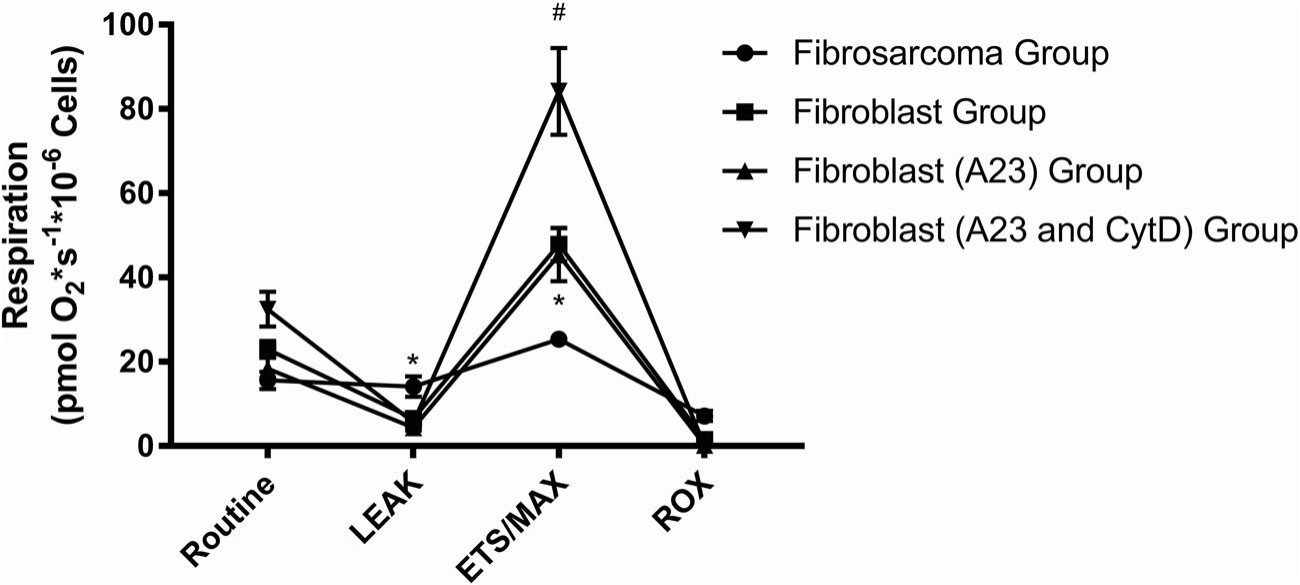
**Respiration in intact cells: cellular mitochondrial respiration obtained in the four groups in key parameters of mitochondrial respiration.** Data are mean±s.e.m. The sarcoma group exhibited higher LEAK state and lower MAX/ETS or uncoupled respiration when compared to the other groups (*P<0.001 for both). The fibroblast (A23 and CytD) group exhibited significantly higher ETS/MAX compared to the other three groups (^#^*P*<0.0001). There were no differences in ROX between the groups. The key parameters in respiration are explained in the Materials and Methods.

We next measured changes in oxygen consumption to assess mitochondrial function in the following four groups: (1) fibrosarcoma group; (2) fibroblast group; (3) fibroblast A23 group; and (4) fibroblast A23+CtyD group. We exposed fibroblasts to A23 alone, similar to in our previous work, to assess the mitochondrial response to calcium entry in addition to treatment of CytD (Kandel et al., 2016). Our previous work demonstrated that the addition of noco+A23 exhibited lower routine respiration, and that increased intracellular calcium with A23 sensitizes cells to decreases in mitochondrial respiration induced with cytoskeletal inhibitors. Fig. 4 illustrates that mitochondrial respiration tracing for the four groups with intact cells.

The fibroblast A23+CytD group exhibited significantly higher routine respiration [pmol*sec^−1^*10^−6^ cells)] (32.5±2.3, *P*<0.05) than either the fibroblast A23 group alone or the fibrosarcoma group. Routine respiration was not significantly higher for the fibroblast group as compared to the fibrosarcoma group (*P*=0.08); however the fibrosarcoma group exhibited a higher LEAK state, at 14.11±2.97, than the fibroblast group treated with A23 alone (5.63±2.97) and the fibroblast group treated with both A23 and CytD (4.35±2.97). The ETS or MAX respiration of the fibrosarcoma group was statistically lower than that of the other three groups (25.3±1.9, *P*<0.001). Interestingly the fibroblast A23+CytD group exhibited significantly higher ETS/MAX respiration (84.2±10.3, *P*<0.0001) when compared to the other three groups.

**Fig. 5.**
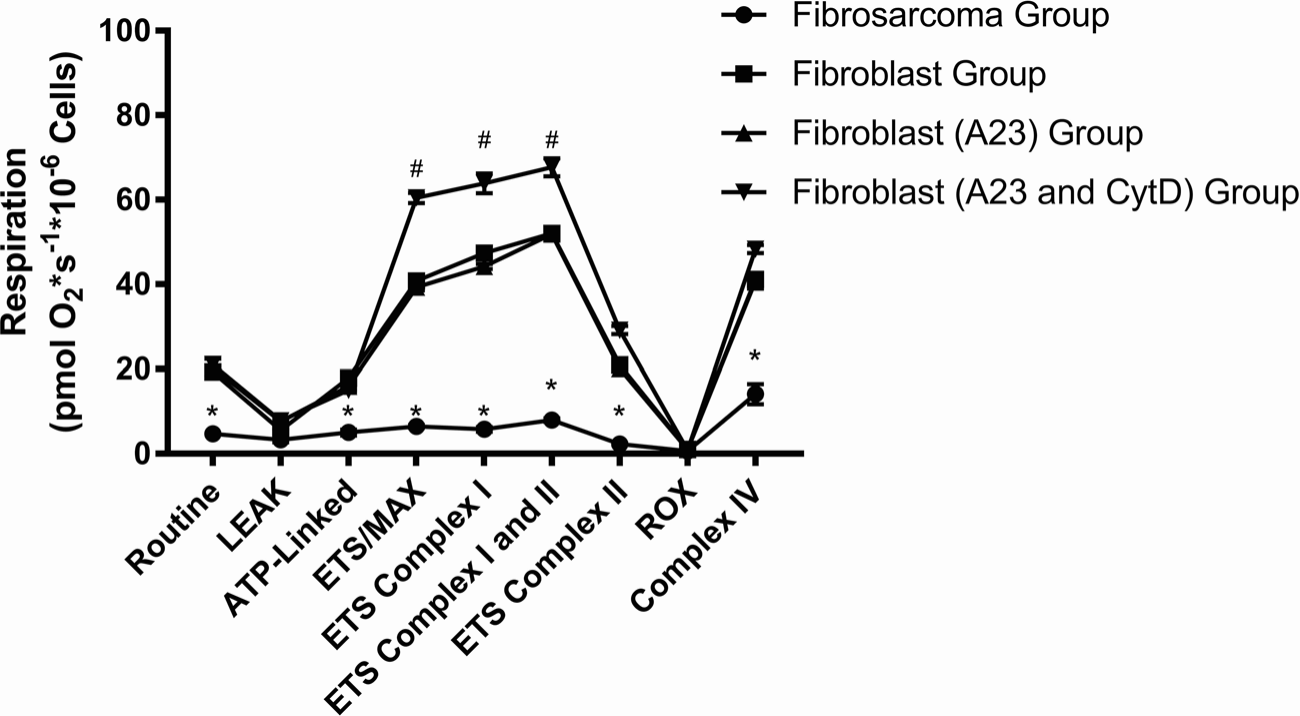
**Complex**-**linked activity in various respiratory states across the four groups.** The sarcoma group exhibited significantly less respiration across all respiratory states, except for LEAK and ROX, compared to all other groups (*P<0.0001). The fibroblast (A23 and CytD) group exhibited significantly higher respiration in the uncoupled state for ETS/MAX and ETS Complex I and I/II when compared to the other three groups (^#^*P*<0.0001). The key parameters in respiration are explained in the Materials and Methods.

We also obtained complex-linked activity with the simultaneous measurement of H_2_O_2_ production using Amplex Red in the four conditions described above after permeabilization of cells. Fig. 5 illustrates complex-linked activity in various respiratory states across the four groups; in general there were low respiration values across different respiratory states for the fibrosarcoma group, which were significantly lower when compared to all the fibroblast conditions in the following respiratory states: Routine (4.7±0.1, *P*<0.0001); ATP-linked (5±0.7, *P*<0.0001); ETS/MAX (6.4±0.3, *P*<0.0001); ETS Complex I (5.7±0.5, *P*<0.0001); ETS Complex I and II (8.0±0.4, *P*<0.0001); ETS Complex II (2.3±0.1, *P*<0.0001); Complex IV (14±2.4, *P*<0.0001). The fibroblast A23+CytD group had significantly higher respiration states when compared to all the other groups in the following: ETS/MAX (60.4±1.2, *P*<0.0001); ETS Complex I (63.9±2.3, *P*<0.0001); and ETS Complex I and II (67.7±2.1, *P*<0.0001).

**Fig. 6.**
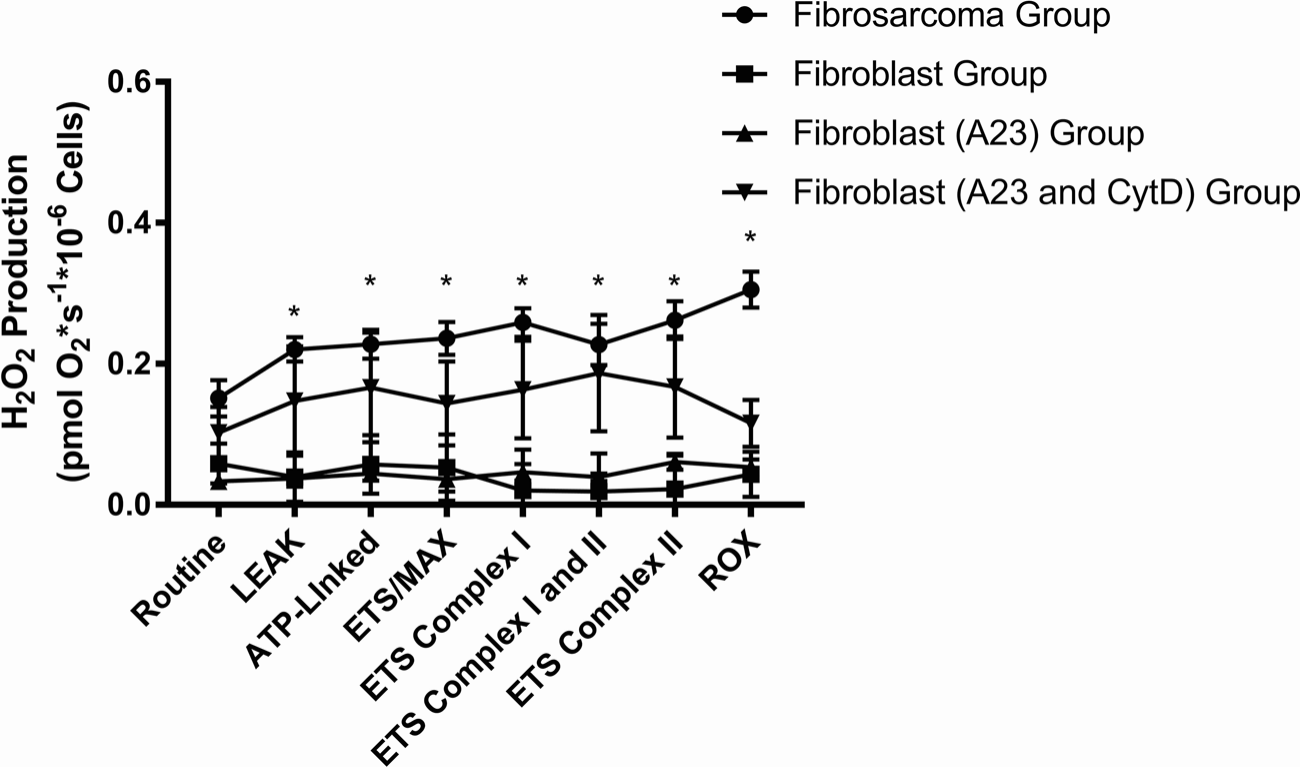
**Changes** i**n H_2_O_2_ production across the groups.** The sarcoma group exhibited overall higher rates of hydrogen peroxide production across all respiratory states except for routine, compared to all other groups (*P<0.0001).

In addition to complex-linked activity in permeabilized cells, we also simultaneously obtained rates of H_2_O_2_ production. Fig. 6 shows the corresponding changes in H_2_O_2_ production. In general, the rates of H_2_O_2_ production were significantly higher in the fibrosarcoma group when compared to both the fibroblast group and the fibroblast A23 group in the following respiratory states: LEAK (0.22±0.02, *P*<0.05); ATP-linked (0.23±0.02, *P*<0.05); ETS/MAX (6.4±0.3, *P*<0.0001); ETS Complex I (0.26±0.5, *P*<0.05); ETS Complex I and II (0.23±0.03, *P*<0.05); ETS Complex II (0.26±0.03, *P*<0.05); residual oxygen consumption (ROX) (0.31±0.03, *P*<0.05).

## DISCUSSION

Our current work illustrates the complex interaction between mitochondrial dynamics and bioenergetics in fibroblast and fibrosarcoma cell lines as well as fibroblasts exposed to the calcium ionophore, A23, and A23+CytD. This study builds on our previous work in which we demonstrated that cytoskeletal impairment directly impacts mitochondrial function in fibroblasts and fibrosarcoma cells. In that work, we used a variety of noco conditions that included noco alone, noco with CytD, noco with A23 and finally noco with CytD and A23. In most of the various noco conditions of our previous study we demonstrated a decrease in routine respiration and MAX respiration. Given that our previous study used a variety of noco conditions, we focused on CytD and A23 (Kandel et al., 2016). Building upon our previous work, we sought to measure changes in mitochondrial dynamics and respiration in response to cytoarchitecture toxins.

In a previous study, we found that actin microfilament depolymerization with CytD increased the net distance traveled by mitochondria, whereas the use of noco resulted in microtubule depolymerization leading to decreased net distance (Kandel et al., 2015). When both the actin network and microtubules in the cell were destabilized, however, net distances traveled by mitochondria were not significantly different than in untreated fibroblasts. Results from our previous study were confirmed in this study, and additional stress caused by adding a calcium ionophore resulted in a further increase in mitochondrial net distances traveled. Because the actin cytoskeleton has been implicated in the immobilization of mitochondria at the cell cortex, destabilizing the cell's actin network likely caused a decrease in fibroblast cells' ability to immobilize mitochondria at sites of high energetic demand (Boldogh and Pon, 2006). As mitochondria act as transporters for calcium, we suggest that treatment with the calcium ionophore A23 caused a further increase in mitochondrial activity and locomotion as a response to the increased presence of calcium in the cell (Bianchi et al., 2004). Mitochondrial dynamics is also regulated by several motor proteins, which include trafficking kinesin proteins (TRAKs). TRAKs play an important role in the movement of mitochondrial to meet the energetic demands of cells, in particular the neurons. Pathogenic variants in TRAKs have been associated in cases of inherited fatal encephalopathy, helping to establish the importance of normal mitochondrial movement in cells (Barel et al., 2017). We attribute the decreased mitochondrial net motion due to destabilization of microtubules to the elimination of kinesin and dynein motors associated with mitochondrial transport.

Untreated fibrosarcoma cells exhibited greater net movement than untreated fibroblasts and their counterpart treatment, likely due to their high demands for energy facilitating rapid cell division. Fibrosarcoma cells treated with noco followed the same pattern as noco treatment in fibroblasts, exhibiting a significant decrease in mitochondrial net distance compared to all other fibrosarcoma conditions. Contrary to the results on fibroblasts, however, CytD and A23 treatments alone did not affect mitochondrial net motion in fibrosarcoma cells, and together caused an overall decrease in mitochondrial net motion. Cancer cells, including fibrosarcoma, differ in many properties when compared to their healthy cell counterparts, including their cytoskeletal architecture and bioenergetic function. For example, noco treatment resulted in increased elastic modulus values in HT 1080 cancer cells whereas no significant difference was found for treated fibroblasts. Thus, it is possible that the increased stiffness resulting from noco treatment in fibrosarcoma cells is the cause of the decrease of mitochondrial movement that occurred. This same result is also reflected in intracellular particle dynamics measurements where the particles decrease in motility within noco-treated fibrosarcoma cells but not noco-treated fibroblasts (Grady et al., 2017). Disruption of microtubules in cancer cells affects both cell elasticity and mitochondrial locomotion (Kandel et al., 2015).

In addition to investigating the effects of disrupting the cytoskeletal architecture on mitochondrial movement, we also examined the effect on rates of fission and fusion events in both cell lines exposed to the cytoskeletal stressors described (Kandel et al., 2015). The process of mitochondrial fusion allows cross complementation of damaged mitochondria to maximize oxidative function in the presence of stress. Mitochondrial fission typically occurs in dividing cells to ensure an adequate amount of mitochondria in each cell. Fission can also occur in the setting of mitochondrial stress in order to segregate damaged mitochondrial with severe defects with eventual mitophagy in some cases. Common mitochondrial stresses include genetic mutations, environmental injury, increased ROS production and cytoskeletal alterations.

The cytoskeletal architecture plays an integral role in the normal shape of the mitochondria acting as an external scaffold and also serves an important function in cellular function that includes mitosis and intracellular movements of other organelles. Depending on the cell type, the mitochondria have a specific distribution within a cell dependent on interaction with the cytoskeletal structures and also have specific dynamic patterns to meet specific needs of the cells (Kuznetsov et al., 2009). In highly active cells such as neurons, the mitochondria are very dynamic and localized to regions requiring high ATP production (Anesti and Scorrano, 2006). This is in stark contrast to the mitochondria found in muscle such as cardiac myocytes where they are more static given the function for contraction that require the mitochondria to be in a more fixed position. Building upon our work examining the changes in mitochondrial movement when treated with cytoskeletal toxins and also additionally challenged with A23, we investigated the changes in both fusion and fission events with these toxins.

Untreated fibrosarcoma cells along with those exposed to CytD exhibited a significantly higher percentage of fission events, whereas in fibrosarcoma cells treated with noco, the percentage of mitochondria undergoing fission events was significantly lower than that of fibroblasts treated with noco. Cancer cells such as the untreated fibrosarcoma cells, due to rapid replication, have been shown to exhibit higher rates of mitochondrial fission events in order to populate new cells with mitochondria (Hirokawa, 1982). Our findings also suggest that depolymerization of actin with CytD had little effect on rates of mitochondrial fission, as they were unchanged when compared to those in untreated fibrosarcoma cells. An interesting finding of our study is that fibrosarcoma cells treated with noco resulted in a decreased rate of mitochondrial fission events, suggesting the importance of maintaining proper microtubule scaffolding for proper fission (Ball and Singer, 1982). It has been shown that agents that disrupt microtubules change the mitochondrial disruption of cells, which can also affect mitochondrial division. Our data would suggest a greater role of microtubules in mitochondrial fission when compared to actin filaments. Notably, CytD has been shown to attenuate elevated fission levels due to administration of mitochondrial inhibitors (De Vos et al., 2005). However, when fusion and fission are originally equilibrated, in the absence of an additional stressor, our results suggest that CytD does not have an extended effect on fission rates.

We also determined the differences in mitochondrial respiration between intact fibrosarcoma cells and fibroblasts, as well as fibroblasts treated with A23 alone and A23 with CytD. The LEAK state of the fibrosarcoma cells was statistically lower compared to that of fibroblasts treated with A23 and both A23 and CytD. The MAX respiration of the fibrosarcoma cells was significantly lower compared to that of all of the fibroblast groups, whereas the MAX respiration of the fibroblasts treated with both A23 and CytD was significantly higher than that in all three groups. This is consistent with other reports that compare cancer cells with their normal counterparts. Cancer cells generate energy primarily through glycolysis, in a process known as the Warburg Effect, in strong preference to oxidative phosphorylation, which is thought to allow rapid growth that also explains the increased rate of mitochondrial fission events observed in our study (Rossignol et al., 2004). One of the key parameters in intact respiration is ATP-linked respiration obtained with oligomycin, which is given to inhibit ATP synthase (Complex V) to induce a LEAK state. The difference between routine and LEAK gives respiration linked to ATP production within the fibrosarcoma group that is significantly lower when compared to that in the other groups, which supports the lack of reliance of oxidative phosphorylation in cancer cells (Puurand et al., 2012).

In this study, we treated fibroblasts with A23 (100 nM) alone and A23 with CytD. In our previous study, we found that fibroblasts treated with varying concentrations of CytD alone resulted in no change in respiration compared to control fibroblasts. However, when cells were additionally challenged with A23, cells exhibited a decrease in routine and MAX respiration due to the combined stressors of both increased intracellular calcium and actin depolymerization. These effects were also observed in cells that received both CytD and noco at relatively high doses. However, respiration was also affected by noco alone. Fibroblasts that were given A23 alone did not exhibit any change in respiration compared to fibroblast control cells, which was observed in our previous work. However, an interesting finding was that fibroblasts that were treated with both A23 and CytD exhibited an increase in routine respiration and MAX respiration. It is possible that this change may be the result of differences in methods of cell-cell communication of attached versus suspended cells. Another possible explanation is that an increase in cellular stress may result in a release of cytochrome c leading to the large increase in respiration observed. We performed Trypan Blue dye exclusion staining with Trypan Blue Solution, 0.4% (Invitrogen) for cell viability before and after placement of cells in the OROBOROS O2K and noted a significant decrease in cell viability at 85% for fibroblasts treated with both A23 and CytD as compared to the other groups, which was >95%. The decrease in cell viability for fibroblasts that received both A23 and CytD may be related in cytochrome release leading to cell death (Tiwari et al., 2001).

We also performed a more comprehensive analysis of various respiratory states in addition to simultaneously obtaining H_2_O_2_ production. This is a novel method that allows the simultaneous evaluation in changes in both respiration and H_2_O_2_ production in response to specific injections of various substrate, inhibitors and uncoupler. While measurement of respiration in intact cells allows for global changes in cellular respiration, controlled permeabilization of cells allow entry of various substrates, such as succinate, which normally do not bypass the cellular membrane. Another advantage of this method is the simultaneous measurement of H_2_O_2_ production with the use of Amplex Red, measured as resorufin. In our study, in addition to measuring respiration in intact cells, we obtained detailed respiration data in each study condition. In the fibrosarcoma group, when compared to the other conditions, there was overall decreased respiration across the various complex states. This detailed analysis provides further insight into specific complex linked activity. This further supports the notion that the fibrosarcoma cell line, as with most cancer cell lines, is primarily driven by glycolysis as opposed to oxidative phosphorylation. The fibrosarcoma group exhibited overall lower respiration compared to the other groups at all the complexes.

The fibroblast (A23 and CytD) group exhibited a significantly higher uncoupled state similar to what was observed in the intact cells, but further measurements in permeabilized cells provide more insight with a slight increase in respiration after uncoupling after injection of both Complex I and Complex II substrates. The addition of both glutamate (after ADP is provided) along with succinate resulted in a minimal increase in respiration that suggests that the observed increase in uncoupling may be related to the treatment with both A23 and CytD as discussed above. It is interested to note that Complex IV respiration was similar across all fibroblast groups that suggest that Complex IV does not play a role with the use of A23 and CytD.

The H_2_O_2_ production data we simultaneously obtained in all four groups provide further insight into the role of ROS (H_2_O_2_) and respiration. One of the advantages of the simultaneous measurement of both respiration and H_2_O_2_ production is to evaluate how changes in H_2_O_2_ production may affect respiration, given that the mitochondria are the major site for both oxygen consumption and ROS production. In our study, all the fibroblast groups (control, A23 and A23 with CytD) exhibited low H_2_O_2_ production. It is unlikely the results seen with increased uncoupling in the fibroblasts treated with A23 and CytD are the result of H_2_O_2_ production and that these drugs result in any appreciable changes in H_2_O_2_ production in general. The fibrosarcoma group did exhibit overall higher H_2_O_2_ production compared to all fibroblast conditions. It is well known that the relationship between ROS and cancerous cells is complex. There is evidence that cancerous cells such as fibrosarcoma cells utilize ROS signals to drive proliferation for tumor progression. This increased ROS production does confer a state of increased oxidative stress that is often a target for clinical therapeutics. Our findings of higher H_2_O_2_ production in the fibrosarcoma cells when compared to their noncancerous counterparts is consistent with the existing literature (Schumacker, 2006).

## MATERIALS AND METHODS

### Cell culture and reagents

The following cell lines were used for this study: adult human dermal fibroblasts between passages 1 and 5 (Lifeline Cell Technology, Walkersville, MD, USA), cultured in FibroLife cell culture media (Lifeline Cell Technology, Frederick, MD, USA) as previously described (Kandel et al., 2015); adult human fibrosarcoma (HT1080) (ATCC, Manassas, VA, USA), cultured in Dulbecco's modified Eagle medium, 10% fetal bovine serum and 1% penicillin streptomycin. All cells were incubated at 37°C in a humidified atmosphere with 5% CO_2_. Cells were grown to confluency and harvested by trypsinization. Each cell line was seeded ∼48 h prior to experiments. All cell lines described were recently authenticated and tested for contamination.

Each cell line was exposed to four different conditions as described below: (1) 2.5 μM CytD, (2) 10 μM noco, (3) 100 nM A23, and (4) 2.5 μM CytD and 100 nM A23. Previously, CytD and noco drug concentrations were determined to be intermediate doses, and an A23 concentration of 100 nM was used as its effect on respiration in absence of another stressor was determined to be insignificant.

For experiments involving measuring mitochondrial net movement and rates of fusion/fission events, cells were plated on MatTek 35-mm glass-bottom dishes (MatTek, Ashland, MA, USA) at a density of ∼25,000 cells/dish. Dishes were coated for 30-40 min with 5 µg/ml fibronectin (BD Biosciences, San Jose, CA, USA) dissolved in PBS prior to cell plating. For the measurement of mitochondrial respiration with HRR, cells were trypsinized and resuspended in their respective medium. A cell count of 3-4 million cells/chamber were placed in the OROBOROS Oxygraph-2K (OROBOROS Instruments, Innsbruck, Austria). Cell count and cell viability were performed with Trypan Blue (0.4%) exclusion staining in the Cell Countess II (ThermoFisher Scientific).

### Determination of mitochondrial dynamics

Changes in mitochondrial net movement and rates of fission/fusion events along with mitochondrial position in single cells were assessed using wide-field fluorescence microscopy as previously described (Kandel et al., 2015). This system employed a QImaging QIClick camera (QImaging, Surrey, BC, Canada) (1×1 102 binning, 1392×1040 pixels) attached to an Olympus IX70 microscope (Olympus, Melville, NY, USA) with an Olympus 40x oil immersion objective lens (Olympus) and Photofluor light source (89 North, Burlington, VT, USA). Computer control of the microscope was facilitated by LUDL programmable filter wheels, shutters, and focus control (LUDL Electronic Products, Hawthorne, NY, USA) and images were collected using IPL 3.7 software (BD, Rockville, MD, USA). For each experiment, cells were visualized using standard TRITC and FITC filters. The day before experiments, cells were transfected with CellLight Mitochondria-GFP, BacMam 2.0 (Life Technologies, Grand Island, NY, USA) at a concentration of 40 particles/cell, and kept in the dark at 37°C. After rinsing, cells were placed in Recording HBSS (HBSS pH 7.4 with 1.3 mM CaCl 2, 0.9 mM MgCl_2_, 2 mM glutamine, 0.1 g/l heparin, 5.6 mM glucose and 1% FBS) for imaging. In each experiment, a selected cell was first imaged in both the mitoGFP channels in recording HBSS. At T=0, recording HBSS was removed from the dish and replaced with either recording HBSS (control) or recording HBSS containing a given cytoskeletal toxin (treatment). In our previous study, we used a DMSO concentration that was equivalent to the highest concentration of drug stock solution for which a morphology effect was observed, and found no observed effects resulted from the DMSO solvent rather than the cytoskeletal drugs (Kandel et al. 2015). Cytochalasin D (CytD) was taken from a stock solution of 1 mM in DMSO and noco was taken from a stock solution of 10 mM in DMSO. For A23, a stock solution of 2 mM was used for this study. The dish was then imaged in both channels for no more than 30 min. Data for each experimental group were collected within 2 days, for a total of 12-28 cells/condition. Effects of cytoskeletal toxins were confirmed in a previous publication using Alexa Fluor 546 phalloidin (Life Technologies; for CytD) and TubulinTracker (Life Technologies; for noco) dyes (Kandel et al., 2016).

Images from the mitoGFP channel were preprocessed in ImageJ (https://imagej.nih.gov/ij/) as previously described (Kandel et al., 2015). Briefly, images were first convolved using a 5×5 edge detection filter. The resulting image was then put through a bandpass filter between 2 and 100 pixels. Finally, the image was manually threshold by eye at a level which removed random background pixels in order to maximize signal-to-noise ratio. Object recognition functions in MATLAB R2010a (MathWorks) were applied to the preprocessed image of each cell at each time point in order to assess mitochondrial net movement along with rates of fission and fusion. The code constructed for this purpose is publicly available online and also described in our publication (Kandel et al. 2015). The net movement for each mitochondrion was obtained by tracking the position of the centroid of each object. When an object from a frame overlapped with multiple objects from the next frame, it was assumed that a fusion event had occurred. Similarly, when multiple objects in a frame overlapped with one object in the previous frame, we assumed a fission event had occurred and the number of mitochondrial objects was increased accordingly. The proportion of mitochondria undergoing fusion and fission were calculated by dividing the number of fusion or fission events by the total number of mitochondria in the cell.

The tracks of the mitochondria were used to calculate MSD, according to the following equation 〈*x*^2^〉=〈(*x(t)−x_0_)*^2^〉. MSD is proportional to the diffusion coefficient, *D*, and this relationship can be written as 〈*x*^2^〉=4Dt^α^, where α is the power law diffusion exponent. A random walk or Brownian type diffusion is indicated by α=1. Anomalous diffusion (where α≠1) can be further classified into subdiffusive (α<1), in which motion is confined, and superdiffusive (α>1), in which motion is likely due to active transport.

### Determination of mitochondrial respiration and H_2_O_2_ production

Cells were placed in a 2-ml chamber at a final concentration of 3-4×10^6^ cells/chamber. Measurement of oxygen consumption was performed at 37°C in a high-resolution oxygraph, OROBOROS Oxygraph-2k, correcting for cell number. Oxygen flux (in pmol O_2_/s/10^6^ cells), which is directly proportional to oxygen consumption, was recorded continuously using DatLab software 6 (Oroboros Instruments). The following sequences of reagents were given in what is referred to as a SUIT (Substrate-Uncoupler-Inhibitor Titration) protocol. SUIT protocols are used to study respiratory control within a single experimental assay for changes in oxygen concentration in the chamber (flux).

Cellular respiration was performed on fibroblast controls, fibroblasts treated with A23 (100 nM), fibroblasts pretreated with A23 (100 nM) followed by CytD (2.5 µM) and fibrosarcoma controls. Prior to beginning respiration measurements, cells were pretreated for 30 min as indicated with A23, CytD or a combination thereof. In the fibroblasts treated with both A23 and CytD, they were pretreated prior to the addition of cytoskeletal toxins with 100 nM A23187 (A23; taken from a stock solution of 2 mM) for 20 min. After 20 min, A23-containing medium was removed and replaced with medium containing both A23 and CytD or noco. Our previous work with DMSO groups included a low DMSO concentration and a high DMSO concentration with no effect on mitochondrial respiration so it was not repeated here. In addition, we performed preliminary work with fibrosarcoma cells exposing them to the above treatment with no change, as these cells are primarily glycolytic and there is no appreciable change to respiration as it low to begin with.

After routine oxygen consumption was recorded for 10 min, the following sequential injections of select compounds were carried out adhering to a SUIT protocol that provides a wealth of information on the key parameters of mitochondrial respiration for intact cells. Oligomycin (1 µg/ml), an ATPase inhibitor, was injected to induce a state 4-like respiration also known as LEAK. LEAK represents the dissipative component of respiration which is not available for performing biochemical work and related to heat production. After, carbonyl cyanide m-chloro phenyl hydrazine or CCCP (0.5 µM steps) was carefully titrated for maximum stimulation of mitochondrial respiration, also known as ETS or MAX. Finally, rotenone (0.5 µM), a Complex I inhibitor, was injected followed by antimycin-A (2.5 µM), a Complex III inhibitor, to obtain the ROX. ROX represents the nonmitochondrial consumption of the cell and was corrected for in this study. Fig. S3 shows a schematic of the injection protocol used.

In addition to performing respiration in intact cells, we also performed a specialized protocol to evaluate specific complex (CI-CIV) activity that requires controlled permeabilization of cells with digitonin, followed by a series of injections of various substrates, inhibitors and uncouplers to obtain a more comprehensive information of individual respiratory states often referred to as reference protocol. In addition to routine, LEAK, ETS/MAX, ROX obtained with intact cells, we also obtained the following respiratory control states: ATP-Linked, Complex I, I and II, and II in the uncoupled state following FCCP to induce ETS/MAX or uncoupling followed by Complex IV respiration. We also performed simultaneous measurement of H_2_O_2_ production with the following injections were performed prior to the injection of cells. First, Amplex Red (10uM) was added followed by horseradish peroxidase (1 U/ml), which is necessary for the conversion of H_2_O_2_ and Amplex Red to resorufin to provide the fluorescence that is detected in various respiratory states based on previous work. This allows measure of H_2_O_2_ production and respiration in various control states as described above. (Jang et al., 2017; Krumschnabel et al., 2015).

### Statistical comparisons

For oxygen consumption measurements, One-way ANOVA followed by Dunnett's multiple comparisons test was performed using GraphPad Prism version 7.00 for Windows (www.graphpad.com). R was then used for adjusting *P*-values for multiple comparisons (p.adjust) using the Benjamini-Hochberg method. Kolmogorov–Smirnov (kstest in MATLAB) was used for the statistical tests for pairwise comparison of mitochondria motility. *P*-values were adjusted to a given control group for all comparisons and considered significant when alpha <0.05.

## Supplementary Material

Supplementary information
