# Supplementary material for: Mitochondrial dynamics and respiration within cells with increased open pore cytoskeletal meshes
Source: Biol Open. 2017 Nov 14;6(12):1831–9. doi: 10.1242/bio.029009 (PMC5769657; doi:10.1242/bio.029009)

Figure S1 illustrates the difference in net movement between the fibroblast and fibrosarcoma groups. The fibrosarcoma cell line exhibits a greater a net movement when compared to the fibroblast cell line, likely due to the increase in dynamic homogeneity in the fibrofibrosarcoma cell line. Fibroblasts exposed to CytD exhibited greater net movement than fibroblast but less than the fibrosarcoma group.

As net distances cannot go below zero, without lognormal distribution the distribution would be skewed to the right.

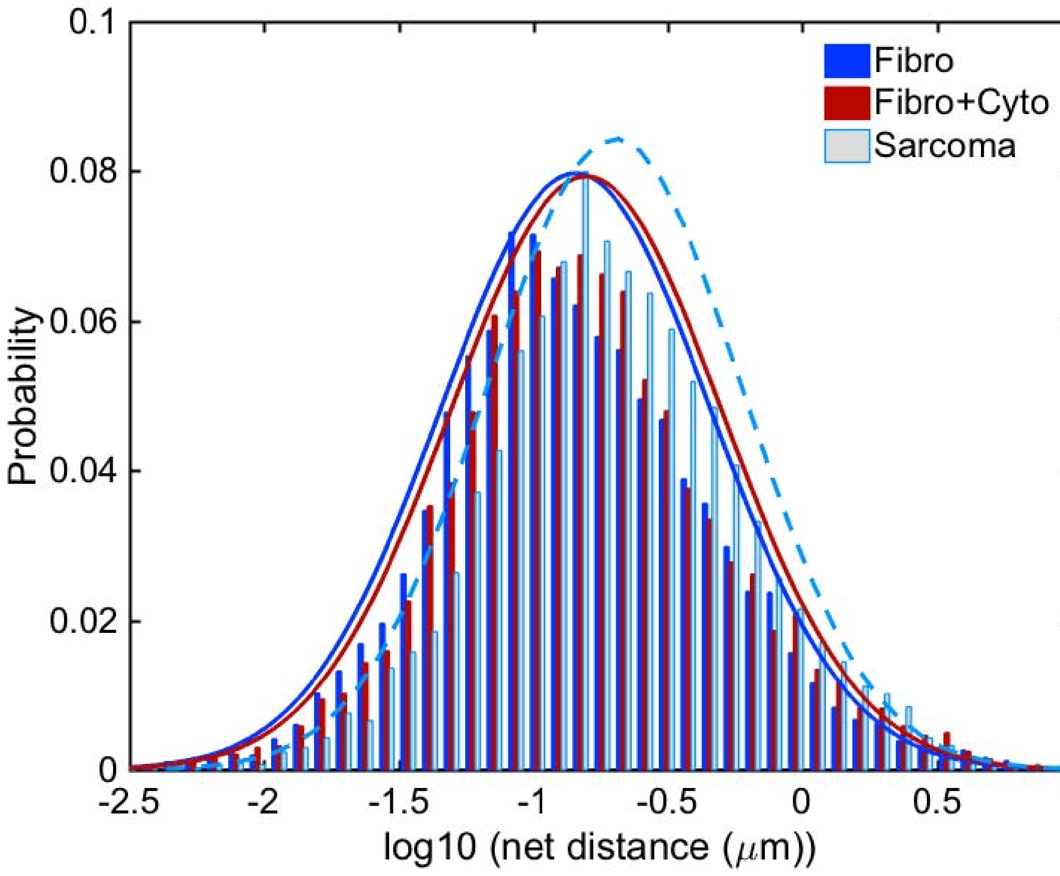

Biology Open • Supplementary information

Table S1

P-value table for total mitochondrial distances traveled.

	FB	FB+cyt	FB+A23	FB+A23+cyt	FB+noco	FS	FS+cyt	FS+A23	FS+A23+cyt	FS+noco	geomean totdist (nm)	Ave velocity (nm/s)
FB	x	-3.059	-8.889	-10.853	-22.558	-11.419	-9.948	-8.992	-6.872	-3.014	5224.8	17.7
FB+cyt	x	x	-19.642	-15.017	-34.858	-6.969	-7.343	-12.985	-12.723	-9.380	5735.7	19.1
FB+A23	x	x	x	-5.669	-17.918	-17.256	-15.311	-9.025	-9.379	-2.917	4740.8	15.8
FB+A23+cyt	x	x	x	x	-40.659	-5.932	-3.548	-1.499	-1.439	-8.387	5077.6	16.9
FB+noco	x	x	x	x	x	-59.005	-57.967	-37.412	-43.239	-9.991	4206.9	14.0
FS	x	x	x	x	x	x	-0.785	-3.151	-2.393	-20.772	5654.2	18.9
FS+cyt	x	x	x	x	x	x	x	-2.538	-1.271	-17.474	5552.9	18.5
FS+A23	x	x	x	x	x	x	x	x	-0.518	-10.800	5205.4	17.4
FS+A23+cyt	x	x	x	x	x	x	x	x	x	-11.832	5245.4	17.5
FS+noco	x	x	x	x	x	x	x	x	x	x	4906.4	16.4

Figure S2: Distribution of power law diffusion exponents of mitochondria within the cell for control fibroblast and fibrosarcoma cell conditions. Both the fibroblast and fibrosarcoma cases display average power law diffusion coefficients of α <1, indicating that mitochondria are subdiffusive within the cell. In fibrosarcoma control cells, however, motion of mitochondria is closer to Brownian.

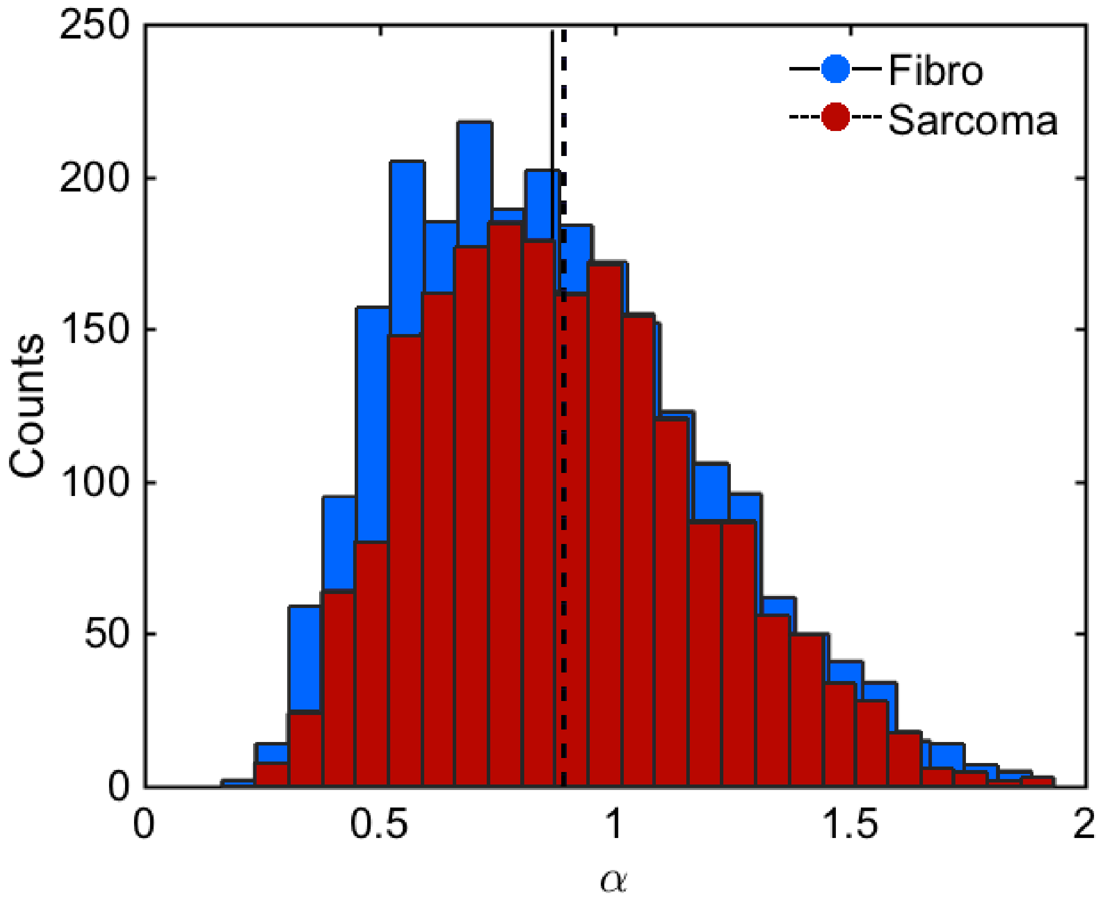

Biology Open • Supplementary information

Table S2: Diffusion coefficients of each cell line and drug condition. Tracks that fit a power law and classified as diffusive were used to calculate the diffusion coefficient.

Cell Type	# tracks	Power law r^2>0.7	Geomean(alphas)	Diffusive Tracks 0.75<alpha<1.25	Diffusion coefficient (um^2/s)
FB	3662	2387	0.809	1108	6.68E-04
FB+cyt	2902	1867	0.771	811	1.34E-03
FB+A23	4613	3197	0.830	1600	8.52E-04
FB+A23+cyt	5593	3665	0.794	1706	1.25E-03
FB+noco	3201	2039	0.729	831	6.53E-04
FS	2972	2013	0.841	1030	1.40E-03
FS+cyt	4100	2951	0.843	1493	1.13E-03
FS+A23	1829	1326	0.865	719	1.04E-03
FS+A23+cyt	3294	2283	0.825	1139	1.42E-03
FS+noco	2810	1930	0.867	885	7.19E-03

Biology Open • Supplementary information

Figure S3: SUIT is the abbreviation for **S**ubstrate-**U**ncoupler-**I**nhibitor **T**itration. SUIT protocols are used with mitochondrial preparations to study respiratory control in a sequence of coupling and substrates states induced by multiple titrations. We used a standard sequence of injections consisting of oligomycin (Omy), CCCP (U), rotenone (Rot), and antimycin (Ama) to study key parameters in mitochondrial respiration.

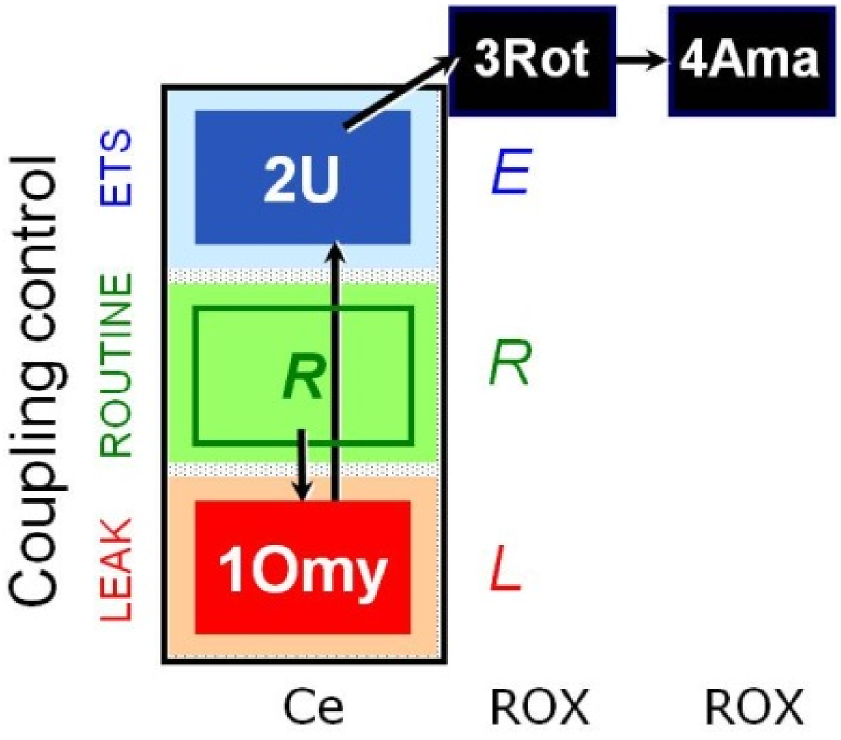

Supplement: Supplementary information [file biolopen-6-029009-s1.pdf]
